# Combined treatment with vitamin C, hydrocortisone and thiamine does not attenuate morbidity and mortality of septic sheep

**DOI:** 10.1186/s42826-024-00213-7

**Published:** 2024-08-12

**Authors:** Tuvshintugs Baljinnyam, Satoshi Fukuda, Yosuke Niimi, Donald Prough, Perenlei Enkhbaatar

**Affiliations:** 1 Department of Anesthesiology, The University of Texas Medical Branch, Galveston, TX USA; 2 Department of Pharmacology and Toxicology, The University of Texas Medical Branch, Galveston, TX USA

**Keywords:** Sepsis, Bacterial clearance, Vitamin C, Hydrocortisone, Thiamine

## Abstract

**Background:**

Sepsis is associated with a highest mortality rate in the ICU. Present study tests the efficacy of combined therapy with vitamin C, hydrocortisone and thiamine (combined therapy) in the ovine model of sepsis induced by *Pseudomonas aeruginosa*. In this study, sepsis was induced in sheep by instillation of *Pseudomonas aeruginosa* (1 × 10^11^ CFU) into the lungs via bronchoscope, under anesthesia. Nine hours after injury, intravenous infusion of vitamin C (0.75 g every 6 h), hydrocortisone (25 mg every 6 h), and thiamine (100 mg every 12 h) or saline was given to the treatment and control groups. Cardiopulmonary variables were recorded.

**Results:**

The survival rate was 16.7% in control and 33.3% in treatment groups. In the control group, mean arterial pressure dropped from 93.6 ± 8.6 to 75.5 ± 9.7 mmHg by 9 h, which was not affected by the combined therapy. Pulmonary dysfunction was not attenuated by the combined therapy either. The combined therapy had no effect on increased extravascular lung water content and fluid effusion into thoracic cavity. The bacterial number in the bronchoalveolar lavage fluid was significantly increased in the treatment group than the control group. The blood bacterial number remained comparable between groups.

**Conclusions:**

Combined vitamin C, hydrocortisone, and thiamine did not attenuate severity of ovine sepsis.

## Background

Sepsis is a disease defined as systemic and deleterious host immune response to infection, and it is associated with highest mortality in the Intensive Care Unit (ICU) worldwide [1]. Infection with antibiotic-resistant microorganisms is attributable to a high mortality of septic patients. One of the opportunistic pathogens is *Pseudomonas aeruginosa*, less frequently found as human microflora in healthy individuals. However, it can lead to sepsis in patients with a broad spectrum of diseases, including burns and respiratory pathologies [2]. In this study, we utilized clinically relevant ovine model for sepsis induced by *Pseudomonas aeruginosa*, because this pathogen was recognized as a main cause of ventilator-associated pneumonia in the ICU [3] and contributes to higher mortality rate of the patients. Previously, we have shown that ovine and human immune responses were comparable to the TLR-4 agonists, lipopolysaccharide and monophosphoryl lipid ALPS [4]. Also, it has also been shown that cardiovascular hemodynamic changes to the LPS were similar in two species [5, 6]. Current standard treatment for sepsis is mostly limited to use of antibiotics, vasopressors, and fluid resuscitation [7, 8]. However, increasing risk of antibiotic resistant pathogen is in concern particularly for hospital acquired pathogens.

More than a hundred phase II and III clinical trials have been performed testing various therapeutic agents including anti-TNF, anti-IL-1R, and anti-LPS agents, corticosteroids, immunoglobulin, and activated protein C in septic patients [9]. However, none of them proved efficacy of the testing compound. Despite many years of intensive research, no drug has been translated to clinical practice for the treatment of sepsis [10–13]. In 2017, Paul et al*.* reported that early administration of intravenous vitamin C, hydrocortisone, and thiamine (further referred to combined therapy) attenuated multiorgan failures and improved survival of septic patients greater than 30% [14].

Vitamin C is a scavenger for free radical and inhibits production of reactive oxygen species [15]. Due to its antioxidant property, vitamin C usage has been considered for sepsis treatment and its positive effect for septic patients have been previously reported [16, 17]. At a low dose, hydrocortisone is used in sepsis mainly to support depleted endogenous steroid function [18, 19]. Thiamine is one of the essential sources of energy production from sugar and indirectly involved in the citric acid cycle [20], and its deficiency leads to lactic acidosis by altering aerobic metabolism. Depletion of thiamine was reported approximately in 20%—70% of the septic patients, depending of criteria to define thiamine deficiency [20, 21]. Therapy with thiamine has previously been reported to be beneficial in sepsis [20, 22].

In contrast to work by Paul et al., recent work by Fujii et al. have reported that combined therapy with vitamin C, hydrocortisone, and thiamine did not lead to a rapid resolution of septic shock and was not superior to hydrocortisone alone [23].

Therefore, to shed some lights into the debated issue, we aimed to investigate the efficacy of the combined therapy (vitamin c, hydrocortisone, and thiamine) in a well-characterized ovine model of sepsis induced by *Pseudomonas aeruginosa*.

## Methods

### Care and use of animals

All animals were cared for according to the approved protocol by the Institutional Animal Care and Use Committee of the University of Texas Medical Branch, and studies were conducted in compliance with the guidelines of the National Institute of Health, and the American Physiological Society for the care and use of the laboratory animals.

### Reagents

Vitamin C (Aspen, #V-0348-05), hydrocortisone (Pfizer, #0009-0825-01), thiamine (Fresenius Kabi, #45816G), and 0.9% sodium chloride (Baxter, #2B1324X), and *Pseudomonas aeruginosa* were purchased (ATCC, #27317).

### Surgical preparation

Female Merino sheep were surgically instrumented a week before the study as previously described [4]. Briefly, under isoflurane anesthesia via endotracheal tube, a Swan-Ganz thermodilution catheter was inserted to the pulmonary artery through the right jugular vein. A polyvinylchloride catheter was placed in the descending aorta via the right femoral artery. A silastic catheter was positioned in the left atrium through the 5th intercostal thoracotomy. Then, sheep were allowed to recover in the Intensive Care Unit.

### Preparation of *Pseudomonas aeruginosa*

Previously prepared glycerol stock of *Pseudomonas aeruginosa* was used throughout the study. After overnight incubation, optical density of the bacterial culture was determined at 550 nm (OD_550_) and colony forming units (CFU) were calculated using the previously obtained formula. Then, the required volume of bacterial culture was spun to obtain the bacterial pellet, that was washed twice with PBS. Finally, 1 × 10^11^ CFU of live *Pseudomonas aeruginosa* was suspended in 30 mL of 0.9% sodium chloride solution per sheep.

### Bacterial instillation to the sheep lungs

On the day of study, randomly chosen sheep were initially anesthetized with intravenous injection of ketamine (500 mg) and isoflurane inhalation (2–5%) via mask and a tracheostomy tube was placed. Under continued anesthesia via tracheostomy tube, thirty milliliters of bacterial suspension were instilled via bronchoscopy to the lungs (10 mL in lower lobe and 10 mL in middle lobe of the right lung and 10 mL in left lung). To ensure effective delivery of bacteria to the lungs, sheep were kept 10 min under anesthesia following instillation.

### Groups allocation and treatment

At each time, a paired (control and treatment) sheep study was performed to ensure the comparable degree of injury. After the bacterial instillation, sheep were randomly allocated into Control (received only saline) and Treatment groups (combined therapy). Treatment was provided as follows: vitamin c, (0.75 g every 6 h), hydrocortisone (25 mg every 6 h), and thiamine (100 mg every 12 h). To enhance the translational aspects, the first dose was given 9 h after injury (approximate time of septic shock onset), where the mean arterial pressure (MAP) dropped 20 points below from baseline value. Treatment doses were calculated and scaled down based on the ratio between the average body weight of human to sheep.

### Cardiopulmonary function assessment

Cardiopulmonary function was assessed by recording hemodynamic variables, such as mean arterial blood pressure (MAP), heart rate (HR), cardiac output (CO), left atrial pressure (LAP), pulmonary arterial pressure (PAP), and central venous pressure (CVP) every 3 h after injury. Systemic (SVRI) and pulmonary (PVRI) vascular resistance indexes were calculated using a standard formula. Pulmonary function was evaluated by blood gas analysis (i.e., arterial and venous PO_2_, PCO_2_, and saturation) (RAPIDPoint 500; Siemens Healthcare, Erlangen, Germany) and pulmonary mechanics variables (i.e., peak and pause airway pressures, and lung compliance) every 3 h. PaO_2_/FiO_2_ ratio and pulmonary shunt fraction was calculated. Arterial blood lactate levels were measured using a blood gas analyzer, as well.

### Blood cell count

A complete blood cell count was determined in arterial blood collected at baseline (BL), and every 3 h thereafter, using a complete blood cell count analyzer (ADVIA120 hematology system, Malvern, PA, USA).

### Determination of bacterial numbers in the circulation

To determine the bacterial number in circulation, 1 mL of venous blood was collected 12 h after injury. Collected samples were cultured on the Trypticase soy-agar plate at 37 degrees, and colony forming units (CFU) were calculated per milliliter of blood with triplicate. At least five independent samples were analyzed.

### Postmortem assays

At the end of the study or when sheep met euthanasia criteria, sheep were humanely euthanized, and half of the right lung tissue was harvested for evaluation of lung water content by the previously described method [24]. The left lung was used for the bronchial alveolar lavage (BAL) to determine bacterial numbers. BAL fluid was harvested after washing the lung with 50 mL of sterile PBS. From the recovered lavage fluid, serial dilutions were made to obtain appropriate colony numbers with triplicated culturing on a trypticase soy agar plate. At least 5 independent samples were analyzed per group.

### Data analysis

Statistical analysis was performed with GraphPad Prism 8. Significance was determined when appropriate with either two-way ANOVA, one-way ANOVA multiple group post hoc test or a two-tailed unpaired Student’s *t *test. *p* < 0.05 was considered to be statistically significant.

## Results

### Administration of the combined drugs

During the administration of the combined therapy no adverse effects were observed. Two out of 6 sheep (33.3%) survived in treatment group vs. 1 out of 6 (16.7%) in the control group survived throughout 24 h study period (*p* = 0.7) (Fig. 5D).

### Hemodynamics

**Fig. 1:**
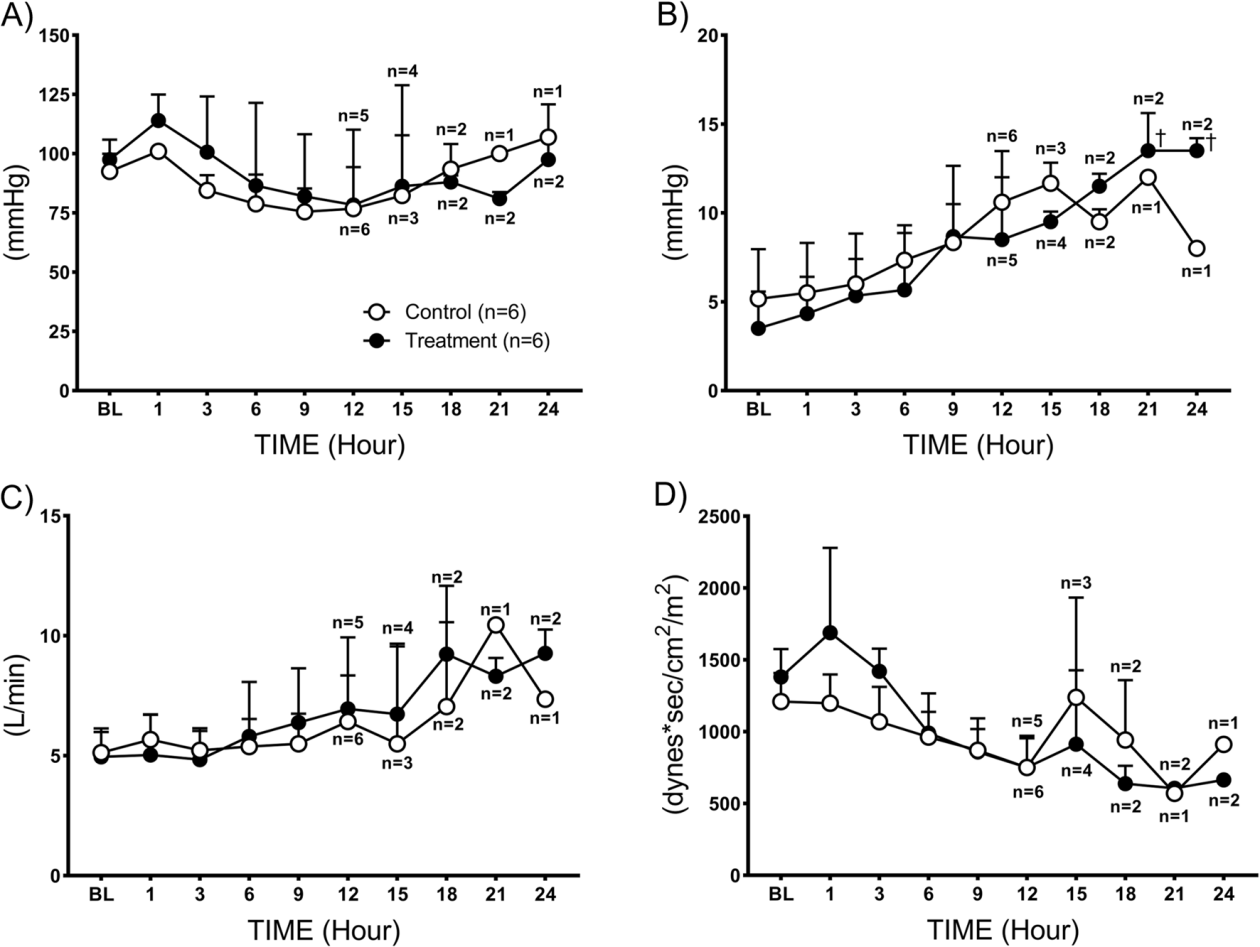
Cardiovascular hemodynamics changes. **A** Mean arterial pressure, **B** central venous pressure, **C** cardiac output, and **D** systemic vascular resistance index. Data are expressed as value ± SD

During the administration of the combined therapy no adverse effects were observed. There were no significant differences in the cardiovascular baseline values between the control and treatment groups. In the control group, MAP was slightly increased at 1 h post injury, and gradually decreased reaching its lowest level at 9 h (75.5 ± 9.7) vs. baseline (92.5 ± 7) (Fig. 1A). CVP gradually increased from the baseline (5.2 ± 2.8) peaking at 15 h (11.7 ± 1.1) in control group (Fig. 1B). Cardiac output tended to increase in both groups compared to the baseline values. No statistical difference was noted (Fig. 1C). SVRI gradually decreased at 12 h (751 ± 220) from the baseline (1210 ± 199) (Fig. 1D) in control group. These hemodynamic changes seen in animals of the control group were not affected by the combined therapy, except for the heart rate that was significantly (*p* = 0.002) higher in the treatment group (Table 1).

**Table 1:** Cardiopulmonary hemodynamics

		Baseline	1 h	3 h	6 h	9 h	12 h	15 h	18 h	21 h	24 h	Significance	*p* value
Animal number at time points	Control	n = 5	n = 6	n = 6	n = 6	n = 6	n = 5	n = 3	n = 2	n = 1	n = 1		
	Treatment	n = 6	n = 6	n = 6	n = 6	n = 6	n = 6	n = 4	n = 2	n = 2	n = 2		
Body temperature	Control	38.9 ± 0.5	40.2 ± 0.5	40.6 ± 0.3	40.3 ± 0.7	39.6 ± 0.5	39.8 ± 0.5	39.7 ± 1.2	40.3 ± 09	40.6	39.9		
	Treatment	38.9 ± 0.6	39.4 ± 0.7	40.4 ± 0.5	40.3 ± 0.7	39.9 ± 0.6	40.1 ± 0.4	40.2 ± 0.7	40.3 ± 0.7	40.6 ± 0.1	40.5 ± 0.2	ns	> 0.05
Heart rate	Control	85.2 ± 16.3	119 ± 28.3	121.3 ± 24.3	117 ± 23	106.7 ± 15.0	108.4 ± 10.1	130 ± 43.3	113 ± 17.0	100	107		
	Treatment	89.3 ± 11.5	102.5 ± 13.5	121.8 ± 12.0	124.5 ± 12.6	120.3 ± 13.8	136.8 ± 8.15†	124.7 ± 23.1	154 ± 17.0†	143.5 ± 2.1†	142 ± 20.0	*	0.002
Cardiac index	Control	5.9 ± 1.2	6.5 ± 0.0	6.0 ± 1.1	6.2 ± 1.5	6.3 ± 1.3	7.2 ± 2.0	6.3 ± 4.8	8.1 ± 4.4	12.3	8.7		
	Treatment	5.6 ± 1.3	5.6 ± 1.6	5.4 ± 1.3	6.4 ± 2.0	7.1 ± 2.6	7.8 ± 3.2	7.3 ± 3.2	9.7 ± 1.4	8.9 ± 0.6	10.1 ± 2.7	ns	> 0.05
Left atrium pressure	Control	9.8 ± 1.5	8.3 ± 2.7	9.6 ± 3.4	11 ± 2.4	15.6 ± 8.9	15.8 ± 8.6	12.3 ± 2.1	14 ± 1.4	13	13		
	Treatment	7.1 ± 2.8	6 ± 2.1	7.8 ± 2.1	9.5 ± 3.6	11.6 ± 4.3	11.8 ± 3.6	14.2 ± 1.2	16 ± 1.4	16 ± 1.4	17 ± 1.4	ns	> 0.05
Hematocrit	Control	27.6 ± 2.4	28.5 ± 1.8	32 ± 1.3	36.3 ± 4.0†	32 ± 3.1	35.3 ± 2.1	35.3 ± 7.6	33.5 ± 9.2	26	25		
	Treatment	27 ± 2.5	32 ± 3.9	34.3 ± 2.5	34.2 ± 4.7†	33 ± 2.8	33 ± 3.9	31 ± 2.2	30.5 ± 0.7	29 ± 2.8	0 ± 0	ns	> 0.05
Lvswi	Control	158.0 ± 24.0	142.3 ± 57.0	103.0 ± 40.3	100.8 ± 45.4	105.1 ± 42.3	125.8 ± 67.2	126.4 ± 121.2	172.4 ± 123.2	118.5	171.0		
	Treatment	158.3 ± 51.1	157.2 ± 45.1	112.4 ± 45.6	119.0 ± 81.3	119.0 ± 73.1	111.1 ± 76.1	121.1 ± 66.1	134.1 ± 17.1	119.1 ± 1.2	171.0 ± 47.0	ns	> 0.05
Tidal volume	Control	520 ± 230	x	532 ± 244	507 ± 214	545 ± 306	596 ± 365	453 ± 217	520 ± 467	370	350		
	Treatment	415 ± 67.1	x	408 ± 67	407 ± 70	416 ± 65	408 ± 67	380 ± 125	440 ± 113	405 ± 35	410 ± 57	ns	> 0.05

Sheep were received to *Pseudomonas aeruginosa* (1 × 10^11^ cfu), and 9 h after treated with either 0.9% sodium chloride (control) or combined therapy (treatment) intravenously. Number of animals per group (n = 6). Values ± standard deviation for body temperature (Celsius), heart rate (BPM), cardiac index (L*min^−1^*m^−2^), left atrium pressure (mmHg), hematocrit (% of blood cell), LVSWI-left ventricular stroke work index (gm-m/m^2^/beat), and tidal volume (ml). The x stands for missing measurement, *p < 0.05 indicates significant difference between groups, and †*p* < 0.05 indicates significant difference compared to baseline

**Fig. 2:**
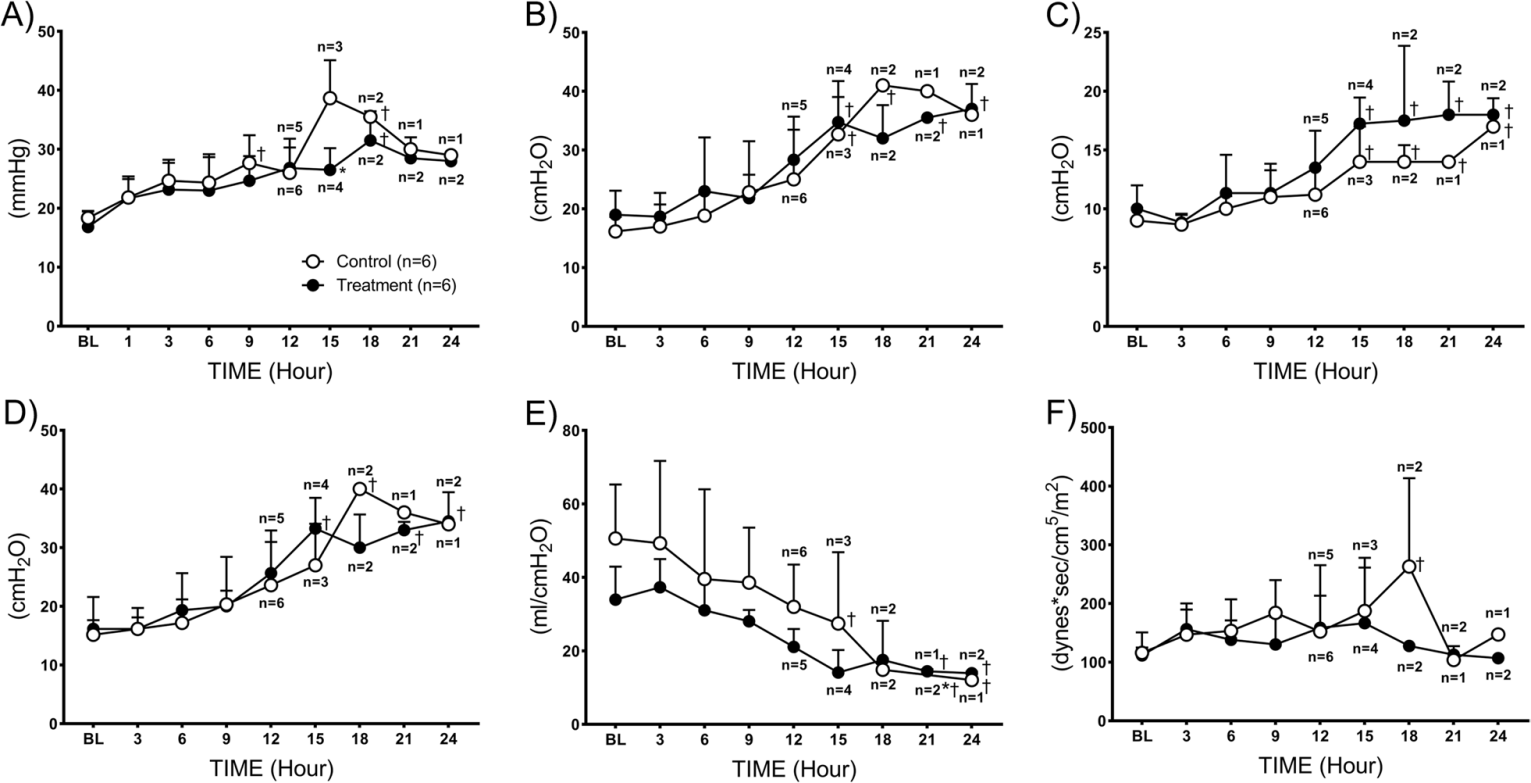
Pulmonary hemodynamics and mechanics. **A** pulmonary arterial pressure, **B** peak airway pressure, **C** mean airway pressure, **D** plateau airway pressure, **E** static compliance, and **F** pulmonary vascular resistance index (PVRI). Data are expressed as value ± SD. *p < 0.05 indicates significant difference between the groups, †*p* < 0.05 indicates significant difference compared to baseline

All baseline values of the pulmonary hemodynamic variables were comparable between the control and treatment groups. In the control group, PAP gradually increased, and the statistically significant increase was observed at 9 h (28 ± 5), and at 18 h (31 ± 5) compared to its baseline (18 ± 1). A similar trend was observed in the treatment group, except the combined therapy significantly reduced (*p* = 0.04) the elevation of PAP at 15 h, which was 26 ± 4 in the treatment and 38.7 ± 4 in control groups (Fig. 2A). In the control group, peak airway pressure gradually increased reaching statistical significance at 15 h (33 ± 9) compared to the baseline (16 ± 2), and a similar trend was observed in the treatment group (Fig. 2B).

Mean airway pressure gradually increased in both groups, reaching statistical significance at 15 h (16 ± 3) and kept higher until 24 h, compared to the baseline (9 ± 1), (Fig. 2C). In the control group, the plateau airway pressure gradually increased, reaching statistical significance at 18 h (40 ± 0) compared to the baseline (15 ± 2). While in the treatment group, it significantly elevated at 15 h (33 ± 5), 21 h (33 ± 1) and 24 h (34 ± 5) compared to the baseline (16 ± 5) (Fig. 2D). There were no statistical differences found between the control and treatment groups.

The static compliance gradually decreased in the control group reaching statistical significance at 15 h (27 ± 19) compared to baseline (51 ± 15), which had a similar trend in the treatment group (Fig. 2E). In the control group, pulmonary vascular resistance index (PVRI) was elevated at 15 h (423 ± 411) and reached statistical significance at 18 h (263 ± 151) compared to the baseline (116 ± 35) (Fig. 2F), while in the treatment group, it stayed at a comparable level to the baseline.

### Pulmonary gas exchange

**Fig. 3:**
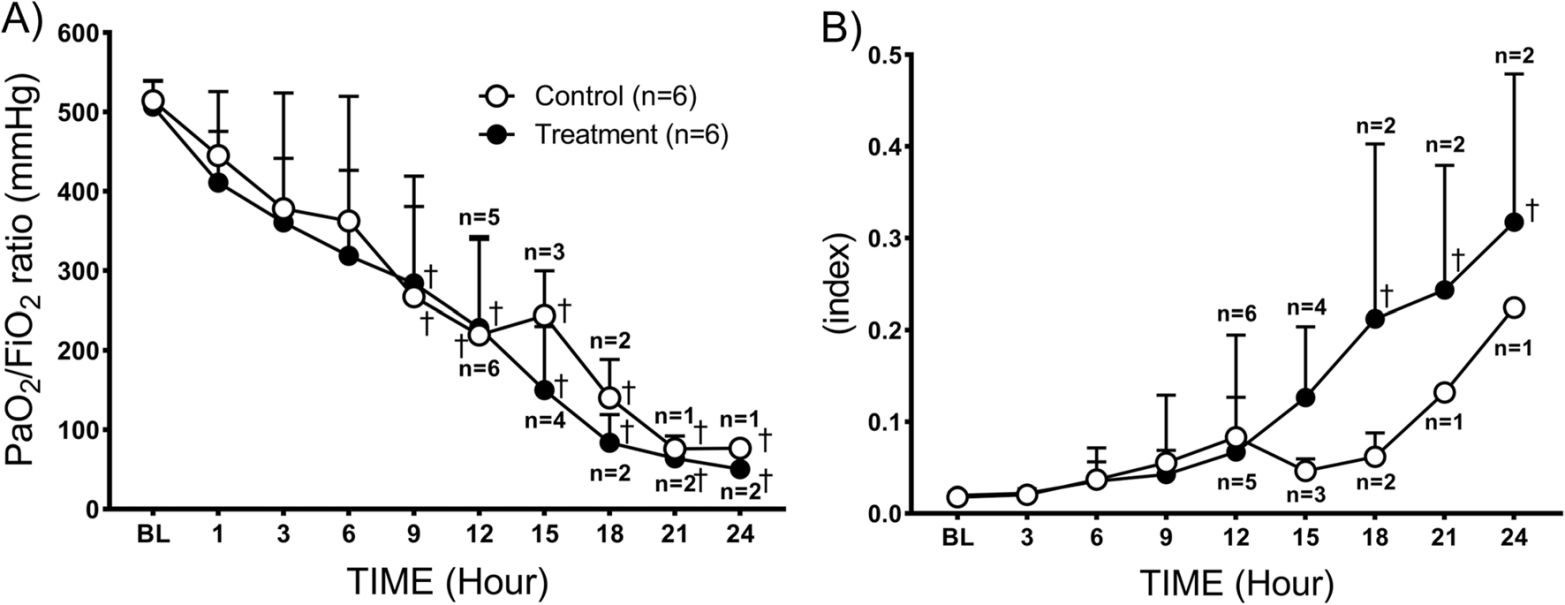
Pulmonary gas exchange. **A** ratio of partial pressure arterial oxygen and inspired oxygen and **B** oxygenation index. Data are expressed as value ± SD

Pulmonary gas exchange gradually deteriorated in both groups throughout the study period. The significant reduction of PaO_2_/FiO_2_ (ratio of the partial pressure of oxygen in the arterial blood and the partial pressure of inspired oxygen) was observed in both the control (267 ± 114) and treatment (284 ± 135) groups at 9 h compared to the baseline (514 ± 25 in control and 507 ± 32 treatment) (Fig. 3A). Administration of combined therapy showed no beneficial effects on pulmonary gas exchange on both PaO_2_/FiO_2_ ratio and oxygenation index (OI) which were comparable in both control and treatment groups (Fig. 3B).

### Microvascular hyper-permeability

**Fig. 4:**
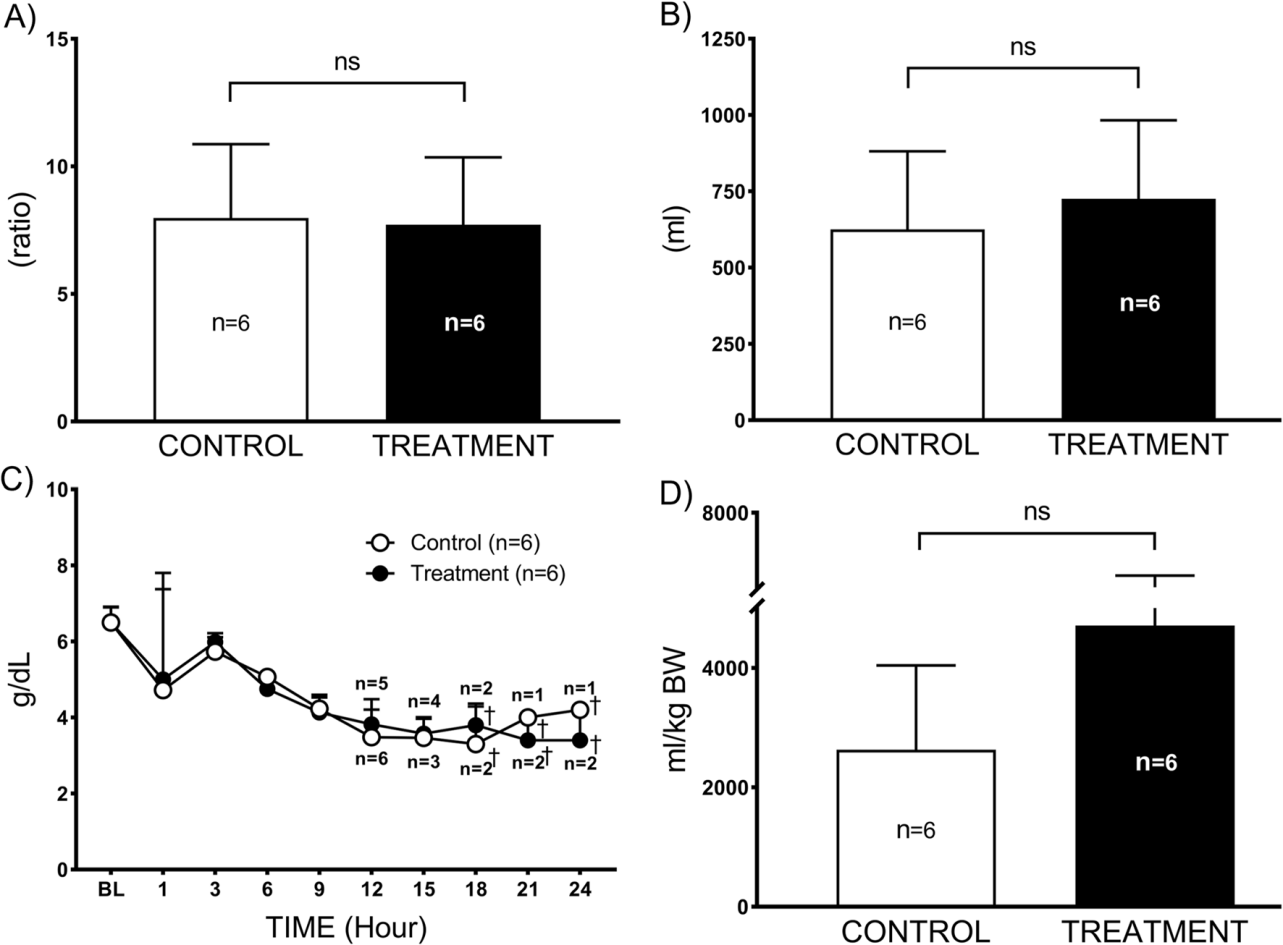
Microvascular hyper-permeability. **A** lung wet-to-dry weight ratio, **B** thoracic fluid accumulation, **C** plasma protein, and **D** accumulated net fluid balance. Data are expressed as value ± SD. †*p* < 0.05 indicates significant difference compared to baseline

Administration of combined therapy showed no beneficial effect on lung water content (*p* = 0.3) evaluated by measuring lung wet-to-dry weight ratio, which was 7.9 ± 2.9 in control and 7.7 ± 2.6 in treatment groups (Fig. 4A). The plasma protein level was significantly decreased at 18 h (3.3 ± 1.0 in control and treatment 3.8 ± 0.6), compared to their baseline (6.5 ± 0.4 in control and treatment 6.5 ± 0.4). No significant differences were noted between the groups (*p* = 0.9) (Fig. 4C). Accumulated net fluid balance increases seen in the control group was not attenuated by the combined therapy (*p* = 0.1) (Fig. 4D). At necropsy, the thoracic fluid level was comparable (*p* = 0.5) between both control (625 ± 256 mL) and treatment groups (726 ± 256 mL) (Fig. 4B).

### Blood cell count

Baselines values for the blood cells counts were comparable between the control and treatment groups. In the control group, the number of white blood cells were sharply decreased reaching the statistically significant difference at 3 h (1276 ± 270) versus the baseline (5480 ± 1119) and remained significantly lower throughout the study compared to the baseline. While eosinophil, polymorphonuclear cell percentage and delta neutrophil index remained at comparable level to the respective baselines. Combined therapy did not affect these variables. Starting at 3 h, neutrophil number sharply decreased (408 ± 208) vs. the baseline (2303 ± 783) and remained significantly lower until 18 h compared to the baseline in the control group. In the treatment group, the neutrophil number was significantly lower throughout the study starting at 3 h (393 ± 114), compared to its baseline (2368 ± 863). A similar trend was observed in the neutrophil percentage changes in two groups (Table 2).

**Table 2:** Blood cell analysis

		Baseline	3 h	6 h	9 h	12 h	15 h	18 h	21 h	24 h	Significance	*p* value
Animal number At time points	Control	n = 5	n = 6	n = 6	n = 6	n = 5	n = 3	n = 2	n = 1	n = 1		
	Treatment	n = 6	n = 6	n = 6	n = 6	n = 6	n = 4	n = 2	n = 2	n = 2		
White blood cell	Control	2303 ± 784	408 ± 208†	593 ± 456†	673 ± 559†	530 ± 588†	280 ± 355†	505 ± 700†	1480	1340		
	Treatment	2368 ± 863	393 ± 114†	528 ± 204†	623 ± 200†	377 ± 244†	220 ± 247†	425 ± 7.07†	375 ± 21.2†	355 ± 120†	*	0.004
Neutrophil	Control	5480 ± 1119	1276 ± 270†	1345 ± 431†	1440 ± 902†	1135 ± 687†	813 ± 503†	1255 ± 940†	2640	2470†		
	Treatment	5932 ± 1431	1463 ± 220†	1403 ± 345†	1438 ± 384†	977 ± 293†	845 ± 514†	1300 ± 113†	1385 ± 233†	1515 ± 601†	ns	> 0.05
Neutrophil (%)	Control	41.3 ± 6.3	36.2 ± 17.5	39.2 ± 19.2	38.1 ± 22.7	37.5 ± 27.1	25.6 ± 20.8	34.0 ± 25.4	56.2	54.3		
	Treatment	39.4 ± 7.4	26.9 ± 7.8	37.1 ± 7.7	43.4 ± 68.5	27.0 ± 15.7	19.2 ± 18.6	32.7 ± 2.05	27.1 ± 2.82	23.8 ± 1.77	*	0.030
Polymorphonuclear cell (%)	Control	46.0 ± 5.2	30.0 ± 3.1	27.9 ± 8.4	35.0 ± 18.3	27.8 ± 10.4	24.4 ± 5.8	25 ± 14.8	30.8	29.8		
	Treatment	42.0 ± 7.8	21.7 ± 8.3	33.2 ± 17.7	25.6 ± 8.4	32.0 ± 9.5	30.4 ± 5.2	17.8 ± 1.1	17.6 ± 7.9	12.5 ± 0.7†	ns	> 0.05
Eosinophil (%)	Control	2.2 ± 1.2	1.8 ± 1.6	2.1 ± 1.2	6.2 ± 6.8	2.6 ± 1.1	5.5 ± 2.5	4.4 ± 0.1	1.9	2.2		
	Treatment	2.3 ± 1.1	1.2 ± 0.3	2.1 ± 2.0	1.8 ± 2.4	2.8 ± 2.5	3.0 ± 0.4	1.6 ± 0.4	1.9 ± 0.8	2.2 ± 0.9	ns	> 0.05
Delta neutrophil index	Control	− 6.9 ± 3.0	4.5 ± 17.2	9.2 ± 21.7	− 3.0 ± 30.1	7.1 ± 20.8	− 4.3 ± 17.5	4.5 ± 10.7	23.5	22.3		
	Treatment	− 4.8 ± 1.5	3.9 ± 6.8	1.8 ± 20.9	16.0 ± 13.4	− 7.7 ± 23.4	− 14.1 ± 19.5	13.3 ± 0.5	7.6 ± 4.2	9.1 ± 3.5	ns	> 0.05

Sheep were received *Pseudomonas aeruginosa* (1 × 10^11^ cfu), and 9 h after treated with either 0.9% sodium chloride (control) or combined therapy (treatment) intravenously. Number of animals per group (n = 6). Values ± standard deviation for white blood cells (cells/uL), neutrophil count (cells/uL), neutrophil percent (%), polymorphonuclear cell percent (%), eosinophil percent (%), and delta neutrophil index (index). *p < 0.05 indicates significant difference between groups, and †*p* < 0.05 indicates significant difference compared to baseline

### Bacterial and lactate clearance

**Fig. 5:**
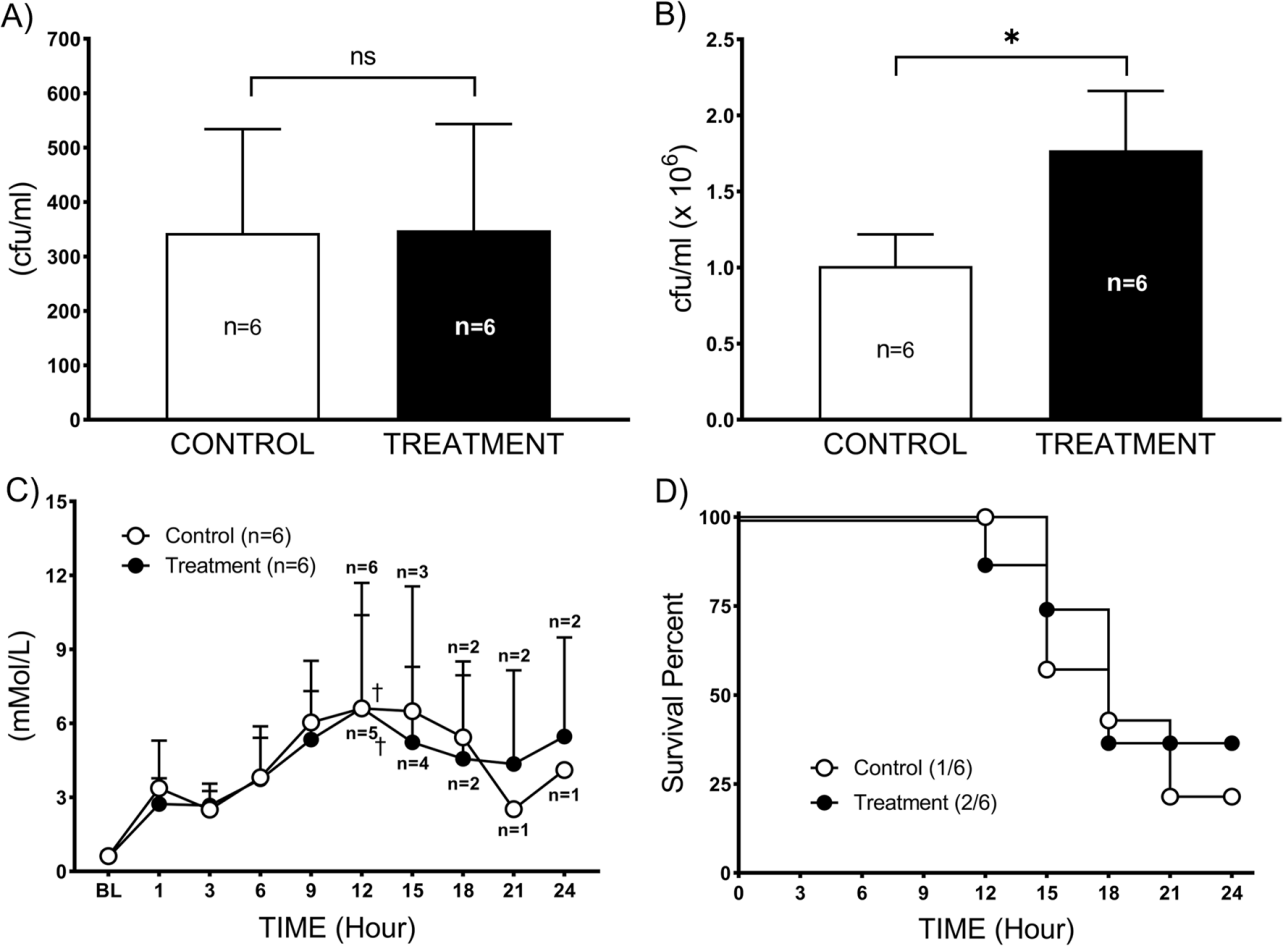
Bacterial clearance and survival. **A** The bacterial number in the circulation and **B** bacterial number in bronchoalveolar lavage fluid (BALF). **C** Lactate level and **D** survival of septic sheep with or without combined therapy. Data are expressed as value ± SD. *p < 0.05 between treatment and control groups. P.A: *Pseudomonas aeruginosa.* †*p* < 0.05 indicates significant difference compared to baseline

Twelve hours after injury, a circulating bacterial number was similar (*p* = 0.9) in both the control (343 ± 190) and treatment (343 ± 191) groups (Fig. 5A). However, bacterial number in BALF was significantly higher (*p* < 0.01) in treatment group (1.7 ± 0.4) vs. control (1 ± 0.2) group (Fig. 5B). The lactate level was similarly increased in both groups, reaching statistical significance in control (6.6 ± 5.1) and treatment (6.6 ± 3.8) at 12 h vs. baseline (0.6 ± 0.2) (Fig. 5C).

## Discussion

In this study we report that combined treatment with vitamin C, hydrocortisone, and thiamine had no effect on multiorgan dysfunctions and the mortality of septic sheep. We have chosen the ovine model as the sheep physiologic [25], hemodynamic [5, 6] and genomic responses to TLR agonists, such as lipopolysaccharide and Monophosphoryl lipid A were reported to be quite similar to those in humans [4].

Previously, it was reported that the combination of vitamin C, hydrocortisone, and thiamine exhibited beneficial effects in septic patients and improved the survival rate by 30% [14]. The authors showed that 28 percent of the patient’s blood was positive for gram positive bacteria (*Escherichia Coli)* and procalcitonin level was > 2 ng/ml [14]. Procalcitonin clearance as well as a sequential organ failure assessment (SOFA) score was improved in these patients by 72 h after treatment [14]. Importantly, the mean duration of vasopressor requirement (hour) was significantly (*p* = 0.001) shortened with the treatment vs. control group.

In contrast, a recent clinical trial by Fujii et al. showed that combined therapy with vitamin C, hydrocortisone, and thiamine did not significantly improve the survival of patients with septic shock [23]. In that study by Fujii et al., patients were included with suspected (or documented) infection and exhibited lactate levels greater than 2 mmol/L. The 90-day survival was 71.4% in control vs. 75.5% in the treatment group. The SOFA score was significantly higher in the treatment group than the control. The vasopressor requirement was comparable in both groups [23]. Despite the similar pulmonary origin of sepsis in both studies [26], the exact reason for the different outcomes is not clear.

Here we report our current study results indicating that the combined therapy exhibits no benefits for cardiopulmonary functions and survival of septic sheep. Combined therapy did not attenuate any measured variables i.e., MAP, pulmonary gas exchange, pulmonary mechanics, lung microvascular permeability, and lactate clearance. Our data supports the outcome of studies by Fujii et al. In the present study, the combined treatment did not affect numbers of circulating bacteria in septic sheep.

It was reported that multiple organ failure inversely correlates with concentration of circulating vitamin C in septic patients [26, 27], and its intravenous supplementation reported to be beneficial in multiorgan functions. Vitamin C supplementation has been shown to reduce endothelial hyper-permeability, improve responsiveness to vasoconstrictors, and scavenge reactive oxygen species [28–31].

Recent evidence indicates the critical role of mitochondrial dysfunction in organ injury during sepsis. As a key cofactor for mitochondrial aerobic respiration and redox status, thiamine has been reported to improve mitochondrial function as evidenced by reduced organ tissue histologic changes and cellular oxygen consumption [32, 33].

Hydrocortisone has been recommended as a useful treatment to decrease mortality in hemodynamically unstable septic shock patients, who are non-responsive to fluids and moderate dose of vasopressor and benefiting from anti-inflammatory and adrenal insufficiency supplementation therapies [34].

To note, in the present study, the bacterial number in the BALF was significantly (*p* = 0.004) higher in the treatment group (1.8 ± 0.4) than the control group (1.0 ± 0.2). As mentioned, we instilled the same amounts of bacteria into the lungs of both groups of sheep conducted side-by-side (treatment and control sheep study). Although, the exact mechanism of the higher number of bacteria in BALF of the treated group remains unclear, our data may suggest that the bacterial clearance might be reduced by the treatment. This may be related to the significant decrease of circulating neutrophils in the treatment group. Our finding is supported by the previous report that the higher mortality of patients treated with vitamin C, hydrocortisone, and thiamine was observed when white blood cell numbers were less than 15.000/mm^3^ [35]. Additionally, we speculate that the combined therapy might support the bacterial infection by sustaining the bacterial propagation. We further speculate that use of hydrocortisone may be associated with immune suppression. Previous studies reported immune suppressive properties of the hydrocortisone at higher dose [1, 36].

Thiamine biosynthesis occurs in bacteria, but not in the human body. During the thiamine synthesis pathway, thiamine monophosphate kinase catalyzes the final step of the pathway by phosphorylating thiamine monophosphate to thiamine pyrophosphate, as the essential cofactor for various crucial cellular processes in bacteria [37]. This may suggest that exogenous administration of thiamine might support the bacterial growth. In regard to vitamin C, we speculate that it may have no effect on the number of BALF bacteria in our present study as it has been shown to inhibit that of *Pseudomonas aeruginosa* biofilm formation without affecting bacterial propagation [38].

Our study has few limitations: (1) Our conclusion on the effects of each tested agents on the bacterial growth and clearance is rather speculative; (2) Efficacy of each individual therapy was not tested; (3) No dose dependent study has been performed for each of the compounds; (4) Effects of the combined therapy was tested only in Gram-negative sepsis; (5) The study duration is relatively short; and (6) Symptomatic therapies, such as antibiotics and vasopressors were not used. Nevertheless, results of our present study demonstrate that a combined therapy failed to attenuate severity of sepsis-associated multiorgan functions and mortality. Our results are supported by results of recent clinical and basic science studies reporting inefficiency of combined therapy with vitamin C, hydrocortisone and thiamine [39–41].

## Conclusions

Based on the obtained results, we conclude that intravenous administration of the combination of vitamin C, hydrocortisone, and thiamine did not exert benefits in the ovine model of sepsis and septic shock induced by *Pseudomonas aeruginosa*.

## Data Availability

Data will be made available on reasonable request.

## Abbreviations

BAL: Bronchial alveolar lavage
BL: Baseline
CFU: Colony forming unit
CO: Cardiac output
CVP: Central venous pressure
HR: Heart rate
MAP: Mean arterial pressure
LAP: Left arterial pressure
PaO_2_/FiO_2_: Ratio of arterial oxygen partial pressure/fractional inspired oxygen
PAP: Pulmonary arterial pressure
PVRI: Pulmonary vascular resistance index
SVRI: Systemic vascular resistance index

## References

[1] Marshall JC, Vincent JL, Guyatt G, Angus DC, Abraham E, et al. Outcome measures for clinical research in sepsis: a report of the 2nd Cambridge colloquium of the international sepsis forum. Crit Care Med. 2005;33(8):1708–16. 16096445 10.1097/01.CCM.0000174478.70338.03

[2] Yang L, Jelsbak L, Marvig RL, Damkiær S, Workman CT, Rau MH, et al. Evolutionary dynamics of bacteria in human host environment. Proc Natl Acad Sci USA. 2011;108(18):7481–6. 21518885 10.1073/pnas.1018249108 PMC3088582

[3] Nseir S, Ader F, Lubret R, Marquette CH. Pathophysiology of airway colonization in critically ill COPD patient. Curr Drug Targets. 2011;12(4):514–20. 21194404 10.2174/138945011794751537

[4] Enkhbaatar P, Nelson C, Salsbury JR, Carmical JR, Torres KE, Herndon D, et al. Comparison of gene expression by sheep and human blood stimulated with the TLR4 agonists lipopolysaccharide and monophosphoryl lipid A. PLoS ONE. 2015;10(12):e0144345. 26640957 10.1371/journal.pone.0144345 PMC4671644

[5] Murakami K, Bjertnaes LJ, Schmalstieg FC, McGuire R, Cox RA, Hawkins HK, et al. A novel animal model of sepsis after acute lung injury in sheep. Crit Care Med. 2002;30(9):2083–90. 12352045 10.1097/00003246-200209000-00022

[6] Enkhbaatar P, Joncam C, Traber L, Nakano Y, Wang J, Lange M, et al. Novel ovine model of methicillin-resistant *Staphylococcus aureus*-induced pneumonia and sepsis. Shock. 2008;29(5):642–9. 17885644 10.1097/SHK.0b013e318158125b

[7] Rhodes A, Evans LE, Alhazzani W, Levy MM, Antonelli M, Ferrer R, et al. Surviving sepsis campaign: International guidelines for management of sepsis and septic shock: 2016. Intensive Care Med. 2017;43(3):304–77. 28101605 10.1007/s00134-017-4683-6

[8] Evans L, Rhodes A, Alhazzani W, Antonelli M, Coopersmith CM, French C, et al. Surviving sepsis campaign: international guidelines for management of sepsis and septic shock 2021. Intens Care Med. 2021;47(11):1181–247. 10.1007/s00134-021-06506-y PMC8486643 34599691

[9] Marshall JC. Why have clinical trials in sepsis failed? Trends Mol Med. 2014;20(4):195–203. 24581450 10.1016/j.molmed.2014.01.007

[10] Hotchkiss RS, Coopersmith CM, McDunn JE, et al. The sepsis seesaw: tilting toward immunosuppression. Nat Med. 2009;15(5):496–7. 19424209 10.1038/nm0509-496 PMC3786779

[11] Hotchkiss RS, Opal S. Immunotherapy for sepsis–a new approach against an ancient foe. N Engl J Med. 2010;363(1):87–9. 20592301 10.1056/NEJMcibr1004371 PMC4136660

[12] Wiersinga WJ. Current insights in sepsis: from pathogenesis to new treatment targets. Curr Opin Crit Care. 2011;17(5):480–6. 21900767 10.1097/MCC.0b013e32834a4aeb

[13] Mitka M. Drug for severe sepsis is withdrawn from market, fails to reduce mortality. JAMA. 2011;306(22):2439–40. 22166598 10.1001/jama.2011.1755

[14] Marik PE, Khangoora V, Rivera R, Hooper MH, Catravas J. Hydrocortisone, vitamin c and thiamine for the treatment of severe sepsis and septic shock: A retrospective before-after study. Chest. 2017;151(6):1229–38. 27940189 10.1016/j.chest.2016.11.036

[15] Cathcart RF. Vitamin C: the nontoxic, nonrate-limited, antioxidant free radical scavenger. Med Hypothesis. 1985;18(1):61–77. 10.1016/0306-9877(85)90121-5 4069036

[16] Tanaka H, Matsuda T, Miyagantani Y, Yukioka T, Matsuda H, Shimazaki S. Reduction of resuscitation fluid volumes in severely burned patients using ascorbic acid administration: a randomized, prospective study. Arch Surg. 2000;135(3):326–31. 10722036 10.1001/archsurg.135.3.326

[17] Fowler AA 3rd, Syed AA, Knowlson S, Sculthorpe R, Farthing D, DeWilde C, et al. Phase I safety trial of intravenous ascorbic acid in patients with severe sepsis. J Transl Med. 2014;12:32. 24484547 10.1186/1479-5876-12-32 PMC3937164

[18] Annane D, Bellissant E, Bollaert PE, et al. Corticosteroids in the treatment of severe sepsis and septic shock in adults: a systematic review. JAMA. 2009;301(22):2362–75. 19509383 10.1001/jama.2009.815

[19] Bollaert PE, Charpentier C, Levy B, Debouverie M, Audibert G, Larcan A. Reversal of late septic shock with supraphysiologic doses of hydrocortisone. Crit Care Med. 1998;26(4):645–50. 9559600 10.1097/00003246-199804000-00010

[20] Depeint F, Bruce WR, Shangari N, Mehta R, O’Brien PJ. Mitochondrial function and toxicity: role of the B vitamin family on mitochondrial energy metabolism. Chem Biol Interact. 2006;163(1–2):94–112. 16765926 10.1016/j.cbi.2006.04.014

[21] Donnino MW, Andersen LW, Chase M, Berg KM, Tidswell M, Giberson T, et al. Randomized, double-Blind, place-controlled trial of thiamine as a metabolic resuscitator in septic shock: A pilot Study. Crit Care Med. 2016;44(2):360–7. 26771781 10.1097/CCM.0000000000001572 PMC4754670

[22] Manzanares W, Hardy G. Thiamine supplementation in the critically ill. Curr Opin Clin Nutr Metab Care. 2011;14(6):610–7. 21912244 10.1097/MCO.0b013e32834b8911

[23] Fujii T, Luethi N, Young PJ, Frei DR, Eastwood GM, French CJ, et al. Effect of vitamin C, hydrocortisone, and thiamine vs hydrocortisone alone on the time and free of vasopressor support patients with septic shock. JAMA. 2020;323(5):423–32. 31950979 10.1001/jama.2019.22176 PMC7029761

[24] Baljinnyam T, Radnaa E, Niimi Y, Fukuda S, Prough DS, Enkhbaatar P. Cutaneous burn diminishes beneficial effect of intracellular administered mesenchymal stem cells on acute lung injury induced by smoke inhalation in sheep. Burns. 2020. 10.1016/j.burns.2020.05.012. 32513501 10.1016/j.burns.2020.05.012 PMC11676003

[25] Louis D, Robin W, Candice L. Sheep (*Ovis aries*) as a model for cardiovascular surgery and management before, during, and cardiopulmonary bypass. J Am Assoc Lab Anim Sci. 2014;53(5):439–48. 25255065 PMC4181684

[26] Borrelli E, Roux-Lombard P, Grau GE, Girardin E, Ricou B, Dayer J, et al. Plasma concentrations of cytokines, their soluble receptors, and antioxidant vitamins can predict the development of multiple organ failure in patients at risk. Crit Care Med. 1996;24:392–7. 8625625 10.1097/00003246-199603000-00006

[27] Helen FG, Michael JD, Nigel RW. Free Radic Biol Med. 1996;20(1):139–43. 8903690 10.1016/0891-5849(95)02022-5

[28] Eskurza I, Monahan KD, Robinson JA, Seals DR. Effect of acute and chronic ascorbic acid on flow-mediated dilation with sedentary and physically active human ageing. J Physiol. 2004;556:315–24. 14754992 10.1113/jphysiol.2003.057042 PMC1664895

[29] Armour J, Tyml K, Lidington D, Wilson JX. Ascorbate prevents microvascular dysfunction in the skeletal muscle of the septic rat. J Appl Physiol. 2001;90:795–803. 11181585 10.1152/jappl.2001.90.3.795

[30] Tyml K, Li F, Wilson JX. Delayed ascorbate bolus protects against maldistribution of microvascular blood flow in septic rat skeletal muscle. Crit Care Med. 2005;33(8):1823–8. 16096461 10.1097/01.CCM.0000172548.34622.DE

[31] Tyml K, Li F, Wilson JX. Septic impairment of capillary blood flow requires nicotinamide adenine dinucleotide phosphate oxidase but not nitric oxide synthase and is rapidly reversed by ascorbate through an endothelial nitric oxide synthase-dependent mechanism. Crit Care Med. 2008;36(8):2355–62. 18596627 10.1097/CCM.0b013e31818024f6 PMC2572871

[32] Lindenbaum GA, Larrieu AJ, Carroll SF, Kapusnick RA. Effect of cocarboxylase in dogs subjected to experimental septic shock. Crit Care Med. 1989;17(10):1036–40. 2791565 10.1097/00003246-198910000-00014

[33] Laurie KS, Grzegorz M. Cellular effects of endotoxin in vitro. I. effect of endotoxin on mitochondrial substrate metabolism and intracellular calcium. Circ Shock. 1983;11(2):85–99. 6357531

[34] Tongyoo S, Permpikul C, Mongkolpun W, Vattanavanit V, Udompanturak S, Kocak M, et al. Hydrocortisone treatment in early sepsis-associated acute respiratory distress syndrome: results of randomized controlled trial. Crit Care. 2016;20(1):329. 27741949 10.1186/s13054-016-1511-2 PMC5065699

[35] Kim WY, Jung JW, Choi JC, Shin JW, Kim JY. Subphenotypes in Patients with Septic Shock Receiving Vitamin C, Hydrocortisone, and Thiamine: A Retrospective Cohort Analysis. Nutrients. 2019;11(12):2976. 31817439 10.3390/nu11122976 PMC6950320

[36] Mishell RI, Lucas A, Mishell BB. The role of activated accessory cells in preventing immunosuppression by hydrocortisone. J Immunol. 1977;119(1):118–22. 326953 10.4049/jimmunol.119.1.118

[37] Kim HJ, Lee H, Lee Y, Choi I, Ko Y, Lee S, et al. ThiL is a valid antibacterial target that is essential for both thiamine biosynthesis and salvage pathway in pseudomonas aeruginosa. JBC. 2020. 10.1074/jbc.RA120.013295. 10.1074/jbc.RA120.013295 PMC7380198 32404369

[38] Pandit S, Ravikumar V, Abdel-Haleem AM, Derouiche A, Mokkapati VRSS, Sihlbom C, et al. Low concentrations of vitamin C reduce the synthesis of extracellular polymers and destabilize bacterial biofilms. Front Microbiol. 2017;8:2599. 29317857 10.3389/fmicb.2017.02599 PMC5748153

[39] Na W, Shen H, Li Y, Qu D. Hydrocortisone, ascorbic acid, and thiamine (HAT) for sepsis and septic shock: a meta-analysis with sequential trial analysis. J Intens Care. 2021;9(1):75. 10.1186/s40560-021-00589-x PMC8684090 34922637

[40] Zayed Y, Alzghoul BN, Banifadel M, Venigandla H, Hyde R, Sutchu S, et al. Vitamin C, thiamine, and hydrocortisone in the treatment of sepsis: a meta-analysis and trial sequential analysis of randomized controlled trials. J Intens Care Med. 2022;37(3):327–36. 10.1177/0885066620987809 33511898

[41] Sevransky JE, Rothman RE, Hager DN, Bernard GR, Brown SM, Buchman TG, et al. Effect of vitamin C, thiamine, and hydrocortisone on ventilator- and vasopressor-free days in patients with sepsis: the VICTAS randomized clinical trial. JAMA. 2021;325(8):742–50. 33620405 10.1001/jama.2020.24505 PMC7903252

